# Restoration of proximal tubule flow–activated transport prevents cyst growth in polycystic kidney disease

**DOI:** 10.1172/jci.insight.146041

**Published:** 2021-05-24

**Authors:** Zhaopeng Du, Xin Tian, Ming Ma, Stefan Somlo, Alan M. Weinstein, Tong Wang

**Affiliations:** 1 Department of C&M Physiology and; 2 Department of Internal Medicine (Nephrology), Yale University, New Haven, Connecticut, USA.; 3 Department of Physiology and Biophysics, Weill Medical College of Cornell University, New York, New York, USA.

**Keywords:** Nephrology, Drug therapy, Mouse models, Transport

## Abstract

Flow-activated Na^+^ and HCO_3_^–^ transport in kidney proximal tubules (PT) underlies relatively constant fractional reabsorption during changes in glomerular filtration rate (GFR) or glomerulotubular balance (GTB). In view of hypothesized connections of epithelial cilia to flow sensing, we examined flow-activated transport in 3 polycystic kidney disease–related mouse models based on inducible conditional KO of *Pkd1*, *Pkd2*, and *Kif3a*. PTs were harvested from mice after gene inactivation but prior to cyst formation, and flow-mediated PT transport was measured. We confirm that higher flow increased both Na^+^ and HCO_3_^–^ absorption in control mice, and we observed that this flow effect was preserved in PTs of *Pkd1^–/–^* and *Kif3a^–/–^*mice. However, flow activation was absent in *Pkd2*^+/–^ and *Pkd2^–/–^* PT. In heterozygous (*Pkd2^+/–^*) mice, a dopamine receptor 1 (DA1) antagonist (SCH23390) restored transport flow sensitivity. When given chronically, this same antagonist reduced renal cyst formation in *Pkd2^–/–^,* as evidenced by reduced kidney weight, BUN, and the cystic index, when compared with untreated mice. In contrast, SCH23390 did not prevent cyst formation in *Pkd1^–/–^* mice. These results indicate that *Pkd2* is necessary for normal GTB and that restoration of flow-activated transport by DA1 antagonist can slow renal cyst formation in *Pkd2^–/–^* mice.

## Graphical abstract

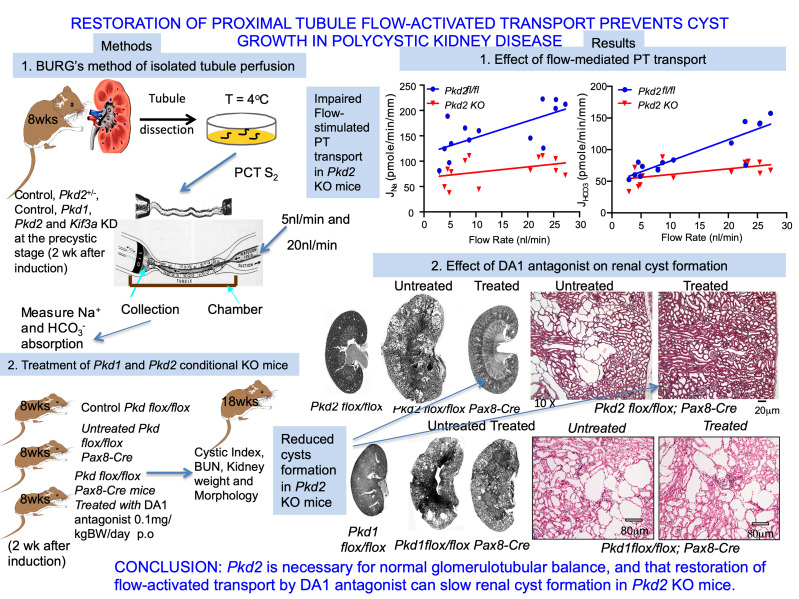

## Introduction

In proximal tubules (PT), flow-modulated salt and water reabsorption is largely responsible for glomerulotubular balance (GTB), namely the constancy of fractional distal Na^+^ delivery during variations in glomerular filtration rate (GFR) (1, 2). Isolated perfused mouse PT have been fruitful for examining the flow dependence of Na^+^ and HCO_3_^–^ transport in vitro. It has been demonstrated that fluid shear stress (FSS) at the luminal surface of the PT cell modulates activity of the Na^+^/H^+^ exchanger (NHE3) and the H^+^-ATPase (3). Available evidence implicates brush border microvilli as the afferent FSS sensors, and analytical predictions of brush border FSS fit experimental observations (4). Signal transduction was actin dependent, since disruption by cytochalasin eliminated flow-dependent changes in transport (5). Flow-stimulated Na absorption was abolished completely by inhibition of NHE3, and it was also abolished in NHE3-KO mice. Flow-stimulated HCO_3_^–^ absorption was substantially reduced by NHE3 inhibition, and it was completely abolished by inhibiting both NHE3 and H-ATPase (3). Specifically, luminal membrane FSS stimulates NHE3 trafficking to the apical membrane by a mechanism that requires an intact actin cytoskeleton, while H^+^-ATPase trafficking depends on the microtubules (6). Cell volume integrity is preserved during flow-dependent transport, since luminal FSS also modulates Na^+^/K^+^-ATPase translocation to the peritubular membrane (6). An important parameter of FSS-dependent Na^+^ reabsorption is the flow sensitivity, defined as the fractional change in transport relative to the fractional change in FSS. With respect to the important regulators of PT transport, angiotensin acts to increase absolute PT Na^+^ and HCO_3_^–^ reabsorption with little perturbation of their flow sensitivity (7). Conversely, dopamine does little to perturb baseline fluxes but markedly blunts flow sensitivity. In these experiments, the dopamine receptor 1 (DA1) antagonist SCH23390 increased flow sensitivity of Na^+^ and HCO_3_^–^ reabsorption to supernormal values (8).

*Pkd1* and *Pkd2* are both localized in primary cilia and also in other cellular locations (9, 10). Polycystin-1 (PC1), the *Pkd1* gene product, is a 4302–amino acid protein consisting of a large, 3000–amino acid extracellular domain; 11 transmembrane domains; and an intracellular carboxyl terminus. It may function as a mechanosensor or chemosensor (10). Polycystin-2 (PC2), the *Pkd2* gene product, is a nonselective calcium permeable cation channel belonging to the TRP channel family. The cellular locations of PC2 have been reported at the cilia and ER, and they can function as Ca^2+^ channels in both locations (11–15). Patch clump recordings identify PC2 as an essential ion channel subunit in the primary cilia of the renal collecting duct epithelium (15). The functional role of the primary cilia has been studied by using cultured MDCK cell and in perfused collecting tubules. It was demonstrated that calcium signals are the major second messenger mechanisms for primary cilium–mediated mechanosensation in MDCK cells and in cortical collecting ducts (4, 16). It was also demonstrated that high flow rate increased intracellular Ca^2+^ concentration and is attenuated in the collecting duct of monocilium-impaired orpk mice (17). Kinesin-like protein (Kif3a), also known as microtubule plus end-directed kinesin motor 3A, is responsible for plus end-directed microtubule sliding activity and is essential for cilia formation. The tissue-specific inactivation of Kif3a in renal tubular epithelial cells results in viable offspring with normal-appearing kidneys at birth, but renal cysts begin to develop in the kidney at P5 and cause renal failure by P21 (18). We have used Kif3a-KO mice, which were generated by flox/flox crossed with pax8-rtTA and Tet-O-Cre mice. Pax8-rTA is a whole nephron Cre (19), and the Tet-O is a Cre with tetracycline responsive element (20).

Previously, we have investigated the impact of calcium signals in the regulation of GTB and have shown that IP3 receptor–mediated intracellular Ca^2+^ signals were critical for transduction of microvillus torque to increase Na^+^ and HCO_3_^–^ absorption (21). It has been reported that primary cilia has a mechanosensiry function in collecting ducts, mediated by increases in intraculluar Ca^2+^ (4, 16, 17). However, whether PC1, PC2, or primary cilia have mechanosensory roles in flow modulation of PT transport has not been examined. Availability of the kidney-selective KO of *Pkd1*, *Pkd2*, and *Kif3a* in animals provides unique tools for investigating whether these molecules are important for mechanosensation in flow-mediated PT transport. We investigated the flow-dependent PT transport in 3 major polycystic kidney disease (PKD) and ciliopathy animal models of *Pkd1-*, *Pkd2-,* and *Kif3a*-KO mice after gene KO but prior to cyst formation. We found that flow dependence of PT reabsorption was absent in *Pkd2^+/–^* and *Pkd2^fl/fl^;Pax8-rtTA;Tet-O-Cre* precystic mice but was preserved in both *Pkd1^fl/fl^;Pax8-rtTA;Tet-O-Cre* and *Kif3a^fl/fl^;Pax8-rtTA;Tet-O-Cre* mice.^.^ In tubules from *Pkd2^+/–^* mice, the dopamine antagonist SCH23390 restored transport sensitivity to flow. When SCH23390 was administered chronically, renal cyst formation was slowed only in *Pkd2^fl/fl^;Pax8-rtTA;Tet-O-Cre* mice but not in *Pkd1^fl/fl^;Pax8-rtTA;Tet-O-Cre* mice. When examined in a mathematical model of the full kidney, abrogation of PT flow–dependent transport produced wider swings in intratubular pressure in conjunction with GFR variations. Our experimental data show that applications of a DA1 antagonist at the precystic stage prevented renal cyst formation only in *Pkd2^–/–^*, which had impaired GTB, but not in *Pkd1^–/–^*, which had intact GTB. These are the results that would be expected if swings in intratubular pressure played a pathophysiological role in cyst formation of *Pkd2*.

## Results

### Flow-stimulated Na^+^ and HCO_3_^–^ absorption in Pkd2^+/–^ and Pkd2^–/–^ mice PT.

Age-matched (8-week-old) male and female mice were used for microperfusion experiments. *Pkd2*^+/–^ and its WT control were generated as described previously (22). The *Pkd2^–/–^* mouse — *Pkd2^fl/fl^;Pax8-rtTA;TetO-cre*, which had received doxycycline induction from P28–P42 — and its control is *Pkd2^fl/fl^* (20) were used for the study. We observed no morphological changes of the kidneys from the *Pkd1-*, *Pkd2-*, and the *Kif3a*-KO mice 2 weeks after the induction (at the age of 8 weeks) and observed some dilated kidney tubules after 5 weeks of induction; renal cysts developed from 8 weeks of induction in *Pkd1* and *Pkd2* KO mice (20). The precystic mice were 8 weeks old (2 weeks after the induction) and were used for the microperfusion experiments.

Table 1 describes the geometry of tubules from all studies, perfused at either low or high flow rates. The inner diameter (ID) and outer diameter (OD) of the perfused tubule under low and high flow rates were measured. Subtraction of the cross-sections defined by OD and ID provides an estimate of cell volumes in all studies (5, 8). As shown in Table 1, higher flow rate significantly increased both ID and OD in all groups of mice. The ID was increased from 50% to 70%, and the OD was increased from 5% to 7% in the transitions from low to high flow rates. Presumably, increases in ID and OD with higher perfusion rates reflect increases in luminal hydrostatic pressure so that these diameter increases reflect tubule compliance. Comparison among the groups revealed no compliance differences between controls and the various KO and *Pkd2*^+/–^ groups. In contrast, the cell volumes under low and high flow rates were not significantly different in all groups of mice (Table 1). These results are in agreement with our previous studies that axial flow alters ion transporters on both apical and basolateral membranes and kept cell volumes relatively constant (6, 23). The last column of the table, T/T_r_, is an estimate of the change in luminal drag in going from low to high flow, specifically the relative torque on the brush border microvilli when viewed as levers (3, 5). This drag is approximately proportional to the luminal fluid velocity and, thus, varies directly with flow rate and inversely with the luminal cross-sectional area.

**Figure 1:**
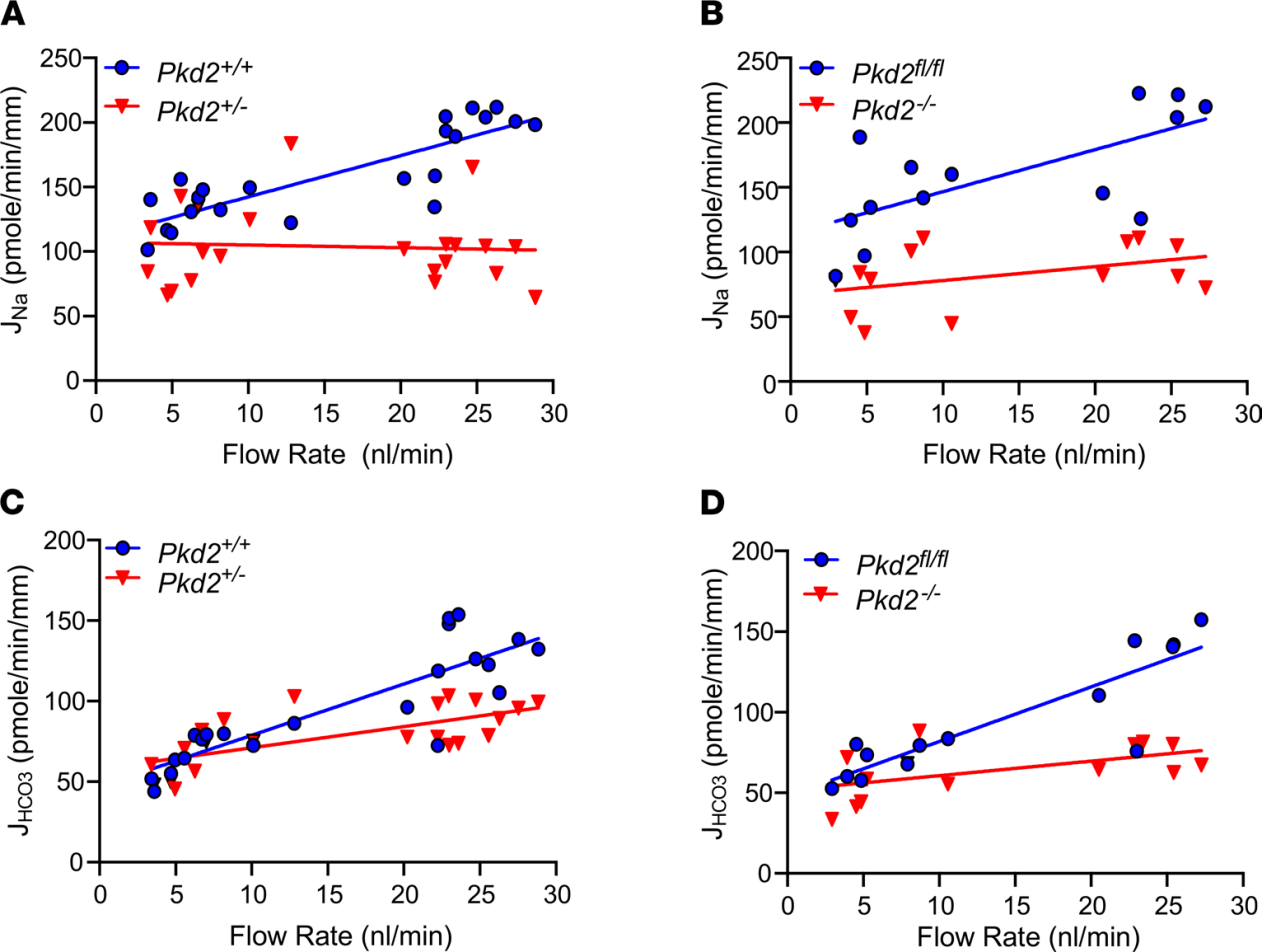
Flow-induced changes in Na^+^ and HCO_3_^–^ absorption in *Pkd2^+/–^* and *Pkd2^–/–^* mice. (**A**–**D**) Flow-induced changes in Na^+^ (J_Na_) and HCO_3_^–^ (J_HCO3_) absorption in control (*Pkd2* WT) and *Pkd2^+/–^* (**A** and **C**), and control (*Pkd2^fl/fl^*) and *Pkd2*^–/–^ (*Pkd2^fl/fl^;Pax8-rtTA;Tet-O-Cre*) (**B** and **D**) mice. PT were perfused in vitro, and tubular fluids were collected under low and high perfusion rates. Each point shows the mean of 3 collections of measurements from the same tubule.

Figure 1, Table 2, Table 3, and Table 4 summarize results of flow-stimulated Na^+^ and HCO_3_^–^ absorption in control, *Pkd2*^+/–^, and *Pkd2*-KO (*Pkd2^fl/fl^;Pax8-rtTA;Tet-O-Cre*) PT. Figure 1A shows the net Na^+^ (J_Na_), and Figure 1C shows the net HCO_3_^–^ (J_HCO3_) absorption in control (*Pkd2* WT) and *Pkd2*^+/–^ mouse PT under low and high flow rates. Similar to results reported previously, J_Na_ increased 47.4% and J_HCO3_ increased 96% in the control group when the flow rate increased from 5.7 nL/min to 24 nL/min (3). The J_Na_ increased from 134.2 to 197.8 pmol/min/mm (*P* < 0.001), and the J_HCO3_ increased from 70.8 to 139 pmol/min/mm (*P* < 0.001) in WT control. In *Pkd2*^+/–^ mice, the flow effect on J_Na_ was completely abolished, and on J_HCO3_, it was considerably reduced (increase of 28%). Figure 1B shows the net Na (J_Na_) and Figure 1D shows the net HCO_3_^–^ (J_HCO3_) absorption in control (*Pkd2^fl/fl^*) and *Pkd2-*KO (*Pkd2^fl/fl^;Pax8-rtTA;Tet-O-Cre*, 2 weeks after the induction). In addition to the slight reduction of J_Na_ at the low flow rate (77.3 versus 137.8 pmole/min/mm; *P* < 0.05) and J_HCO3_ (58.1 versus 74 pmole/min/mm; *P* > 0.05), the flow effect is also completely gone in the *Pkd2-*KO mice compared with the control.

### Flow-stimulated Na^+^ and HCO_3_^–^ absorption in Pkd1- and Kif3a-KO mice PT.

**Figure 2:**
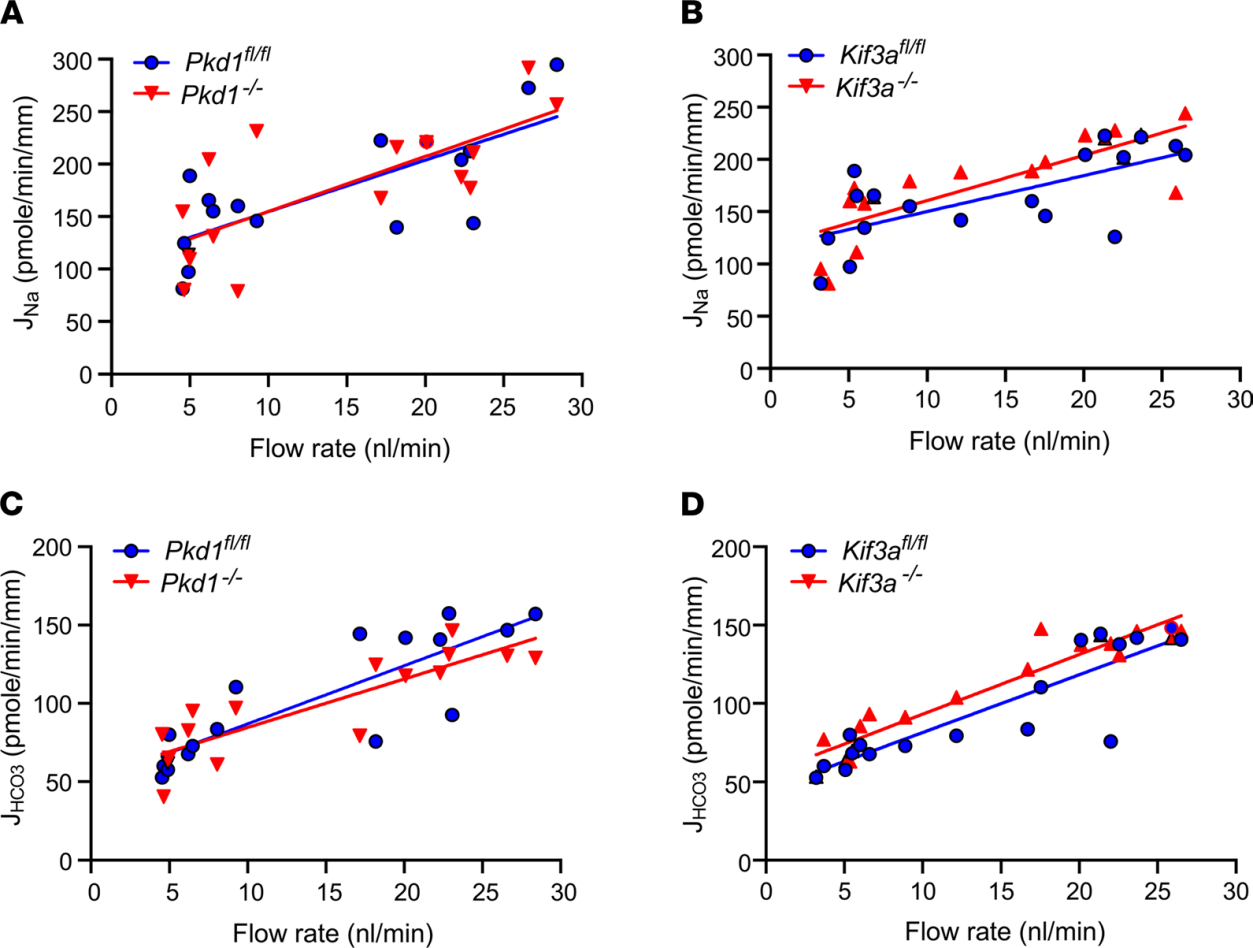
Flow-induced changes in Na^+^ and HCO_3_^–^ absorption in *Pkd1^–/–^* and *Kif3a^–/–^* mice. (**A**–**D**) Flow-induced changes in Na^+^ (J_Na_) and HCO_3_^–^ (J_HCO3_) absorption in control (*Pkd1^fl/fl^*) *and Pkd^–/–^* (*Pkd1^fl/fl^;Pax8-rtTA;Tet-O-Cre*) (**A** and **C**), and control (*Kif3a^fl/fl^*) and Kif3a^–/–^ (*Kif3a^fl/fl^;Pax8-rtTA;Tet-O-Cre*) (**B** and **D**) mice. PT were perfused in vitro, and tubular fluids were collected under low and high perfusion rates. Each point shows the mean of 3 collections of measurements from the same tubule.

Figure 2 summarizes results of flow-stimulated Na and HCO_3_^–^ absorption in control (*Pkd1^fl/fl^*), *Pkd1^–/–^* (*Pkd1^fl/fl^;Pax8-rtTA;Tet-O-Cre*), and *Kif3a^–/–^* (*Kif3a^fl/fl^;Pax8-rtTA;Tet-O-Cre*) mouse PT. KIf3a is a subunit of kinesin-II, which is essential for primary cilia formation. We studied PT functions in *Kif3a^fl/fl^;Pax8-rtTA;Tet-O-Cre* mice at the precystic stage (8-week-old mice, 2 weeks after the induction). Mice at this stage showed absence of primary cilia in the PT, but cysts were not yet formed (20). Figure 2, A and C, shows Na^+^ and HCO_3_^–^ absorption in control (*Pkd1^fl/fl^*) and in *Pkd1^–/–^* mouse PT under low and high flow rates. Increasing flow from 5 to 20 nL/min significantly increased the J_Na_ and J_HCO3_ in control tubules, as we reported previously (3). In *Pkd1^–/–^,* the flow effects are well preserved, and increasing flow rate produced similar stimulation of both J_Na_ and J_HCO3_ in the *Pkd1^–/–^*compared with the control mice. When flow rate increased, J_Na_ increased by 51% and 53% and J_HCO3_ increased by 81% and 99% in control and *Pkd1^–/–^,* respectively (*P* > 0.05). As shown in Figure 2, B and D, flow effects were also well preserved in *Kif3a^–/–^* mice, since similar increments were produced in both J_Na_ and J_HCO3_ by higher flow rates in *Kif3a^–/–^* compared with the control. When flow rate increased, J_Na_ increased by 48% and 45% and J_HCO3_ increased by 84% and 99% in control and *Kif3a^–/–^,* respectively (*P* > 0.05). These results indicate that neither *Pkd1* nor primary cilia are necessary for flow stimulation of Na^+^ and HCO_3_^–^ absorption in PT.

### Effect of dopamine receptor antagonist on flow-stimulated Na^+^ and HCO_3_^–^ absorption.

**Figure 3:**
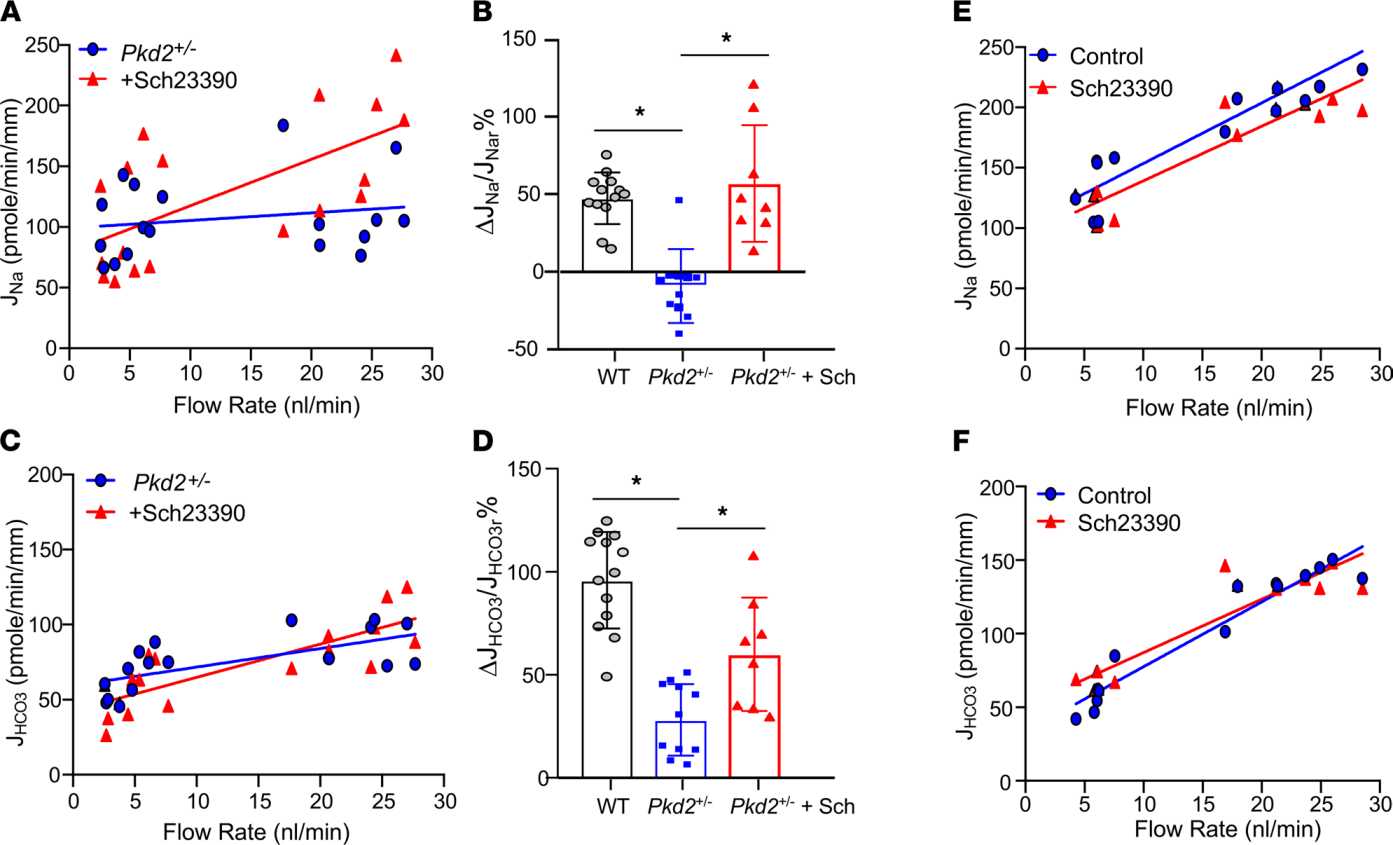
Effect of the dopamine receptor antagonist SCH2339 on flow-stimulated Na^+^ and HCO_3_^–^ absorption. Effect of the dopamine receptor antagonist SCH23390 on flow-stimulated Na^+^ (J_Na_) and HCO_3_^–^ (J_HCO3_) absorption in control (*Pkd2* WT) and *Pkd2^+/–^* mice. PT were perfused in vitro, and tubular fluids were collected under low and high perfusion rates. (**A**, **C**, **E**, and **F**) SCH23390 (1 × 10^–5^M) was added to the luminal perfusate in *Pkd2^+/–^* mice (**A** and **C**) in WT mice (**E** and **F**). (**B** and **D**) ΔJ_Na_/J_Nar_% is the fractional change of differences of high flow rate (20 nL/min) from low flow rate (*r* = 5 nL/min) of Na^+^ and HCO_3_^–^ absorption. *P < 0.05 by 1-way ANOVA; data are presented as the mean ± SEM. J_Nar_, J_Na_ at low flow rate (*r* = 5 nL/min).

Dopamine-stimulated NHE3 endocytosis reduces microvillous expression of NHE3 (24) and blocks the norepinephrine-stimulated Na^+^ transport in PT (25). Previously, we have demonstrated that dopamine inhibits flow-stimulated NHE3 activity by activation of the DA1 receptor (8). Blocking the DA1 receptor by SCH23390 not only restored flow-stimulated transport, but also increased the tubule sensitivity to flow (8). Here, we examined the effect of SCH23390 (1 × 10^–5^M) on flow-stimulated Na^+^ and HCO_3_^–^ absorption in *Pkd2*^+/–^ mice, in which flow-dependent transport is impaired. As shown in Figure 3, flow-stimulated Na^+^ absorption was completely gone (Figure 3A) and HCO_3_^–^ was substantially reduced (Figure 3D) in *Pkd2^+/–^* mice. DA1 inhibition restored the flow effect on Na^+^ and partially restored the flow effect on HCO_3_^–^ in *Pkd2^+/–^* mice. Figure 3, B and D, shows the fractional changes of J_Na_ and J_HCO3_ calculated from the flow rates at 5 to 20 nL/min. The fractional changes in J_Na_ were 47.4%, –9.3%, and 56.8%; the fractional changes in J_HCO3_ were 96%, 28.2%, and 58.6% in WT control, *Pkd2^+/–^*, and *Pkd2^+/–^* + SCH23390, respectively. SCH23390 increased tubule response to flow stimulation on both Na^+^ and HCO_3_^–^ absorption in *Pkd2^+/–^* mice. SCH23390 had no effect on flow-stimulated tubule transport in WT mice. As shown in Figure 3, E and F, the increments in both J_Na_ and J_HCO3_ in the absence or presence of SCH23390 were similar in control tubules.

### Effect of dopamine receptor antagonist on renal cyst formation in Pkd2-KO mice.

**Figure 4:**
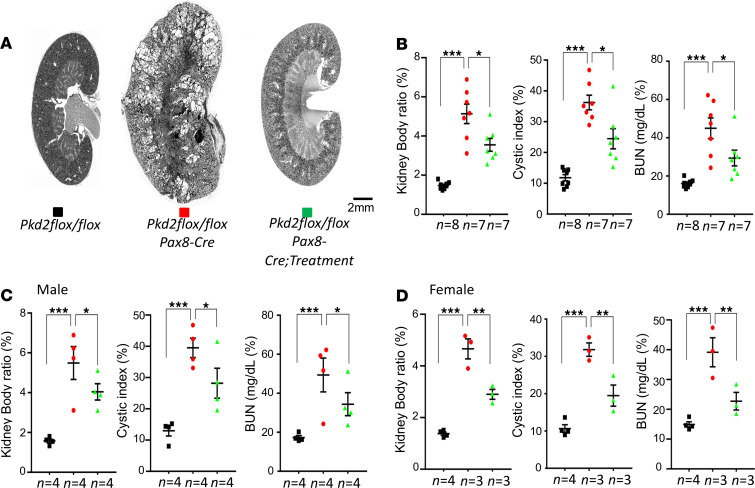
Effect of the dopamine receptor antagonist SCH2339 on renal cysts progression in *Pkd2^–/–^* mice. Inhibition DA1 receptor by SCH23390 can reduce cyst formation in an adult-onset autosomal dominant polycystic kidney disease model with selective inactivation of *Pkd2*. (**A**) Images of representative kidneys from adult-onset KO model (*Pax8 rtTA;Teto-Cre*) with the indicated genotypes at 18 weeks. (**B**) Aggregate data of the kidney weight/body weight ratio, cystic index, and BUN from the indicated number of mice. (**C**) Aggregate data of the kidney weight/body weight ratio, cystic index, and BUN from the indicated number of male mice. (**D**) Aggregate data of the kidney weight/body weight ratio, cystic index, and BUN from the indicated number of female mice. The color blocks in **A** correspond to the data in **B**–**D**. The numbers of animals (*n*) in each group are indicated below the histogram bars. Multiple group comparisons were performed using 1-way ANOVA, followed by Tukey’s multiple comparison test; data are presented as the mean ± SEM.

To study whether a DA1 receptor antagonist can prevent renal cyst formation, the DA1 antagonist SCH23390 was administered in 8-week-old mice through the drinking water (0.1 mg/kgBW/day) for 6 weeks in control (*Pkd2^fl/fl^*) and *Pkd2^–/–^* (*Pkd2^fl/fl^;Pax8-rtTA;Tet-O-Cre*)mice 2 weeks after induction, with doxycycline from p28 to p42. After 6 weeks of treatment, the animals were allowed a 4-week extension for further cystic growth; they were then euthanized at the age of 18 weeks for cyst analysis. The kidney weight/body weight ratio, BUN, and cystic index were analyzed and compared among noncystic control, treated, and untreated *Pkd2^–/–^* kidneys. As shown in Figure 4, the kidney weight/body weight ratio, cystic index, and BUN were elevated in the untreated *Pkd2^–/–^* group compared with the noncystic group. In contrast, the DA1 antagonist SCH23390 reduced kidney weight/body weight ratio, cystic index, and BUN in *Pkd2^–/–^* mice. The cystic index was reduced by 38% (*P* < 0.05) in treated compared with the untreated *Pkd2^–/–^* mice. We also compared the sex differences in response to the SCH23390. As shown in Figure 4, both treated male and female mice had the same reductions on the kidney weight/body weight ratio, cystic index, and BUN compared with untreated mice. Supplemental Figure 1 (supplemental material available online with this article; https://doi.org/10.1172/jci.insight.146041DS1) shows kidney images from all kidney histological sections used in Figure 4. The kidney images showed no cysts in the control, while many cysts formed and the kidney size was enlarged in *Pkd2^–/–^* untreated kidneys. Treated mice had less cystic burden.

**Figure 5:**
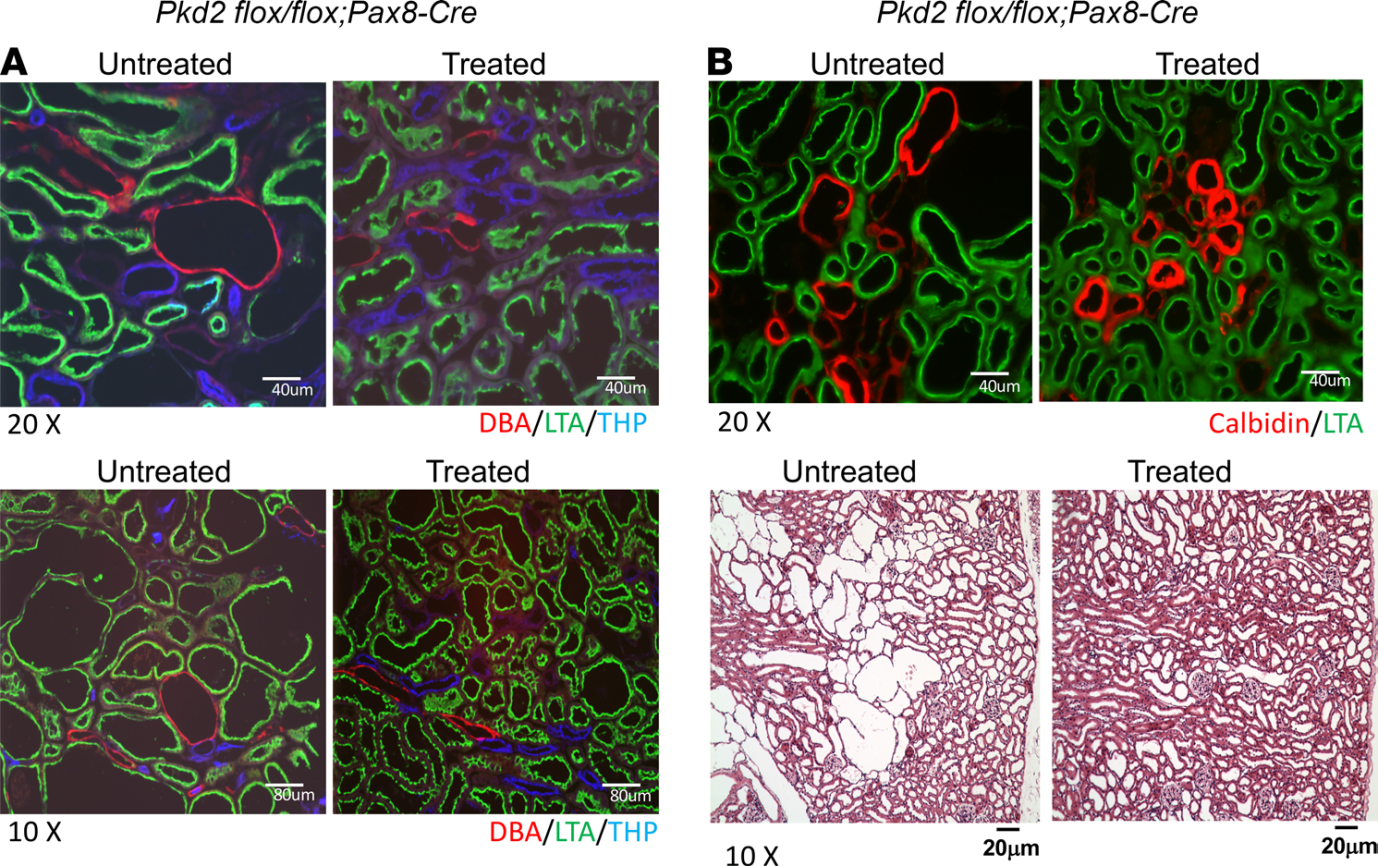
Didney slices with HE staining and IF staining with nephron segmental–specific markers to identify PT, LH, DCT, and CD. (**A**) IF with Rhodamine-conjugated Dolichos biflorus agglutinin (DBA) (red), FITC-lotus tetragonolobus agglutinin (LTA) (green), and sheep anti–tamm horsfall glycoprotein antibody (blue). (**B**) IF with mouse anti-calbidin (red) and FITC-LTA (green). (**C**). Polycystin-2 expression in PT of WT mouse kidney demonstrated with IF staining of rabbit anti–polycystin-2 (green) and FITC-LTA (green). Kidney sections were from 18-week-old adult *Pkd2^fl/fl^;Pax8-rtTA;TetO-cre* mice, SCH23390-treated *Pkd2^fl/fl^;Pax8-rtTA;TetO-cre* and *Pkd2^fl/fl^* (WT) mice that had received doxycycline induction from P28 to P42.

Figure 5 shows kidney slices with H&E staining and immunofluorescence (IF) staining with segmental specific markers of FITC-lotus tetragonolobus agglutinin (LTA); Tamm Horsfall (THP); Calbidinand-conjugated Dolichos biflorus agglutinin (DBA) to identify PT, loop of Henle (LH), Distal convoluted tubule (DCT), and collecting duct. H&E staining shows many tubule cysts and dilated tubules in the untreated kidney section. In contrast, there were only dilated tubules and no large cysts formed in the treated *Pkd2^–/–^* mouse kidney. IF staining with segment-specific markers indicated that dilated tubules and cysts exist in all segments of kidney tubules, and SCH23390 reduced tubule dilation and cysts, formed likely from all segments of the tubules. The reductions are more clearly observed in PT, collecting duct, and DCT.

### Effect of dopamine receptor antagonist on renal cyst formation in Pkd1-KO mice.

**Figure 6:**
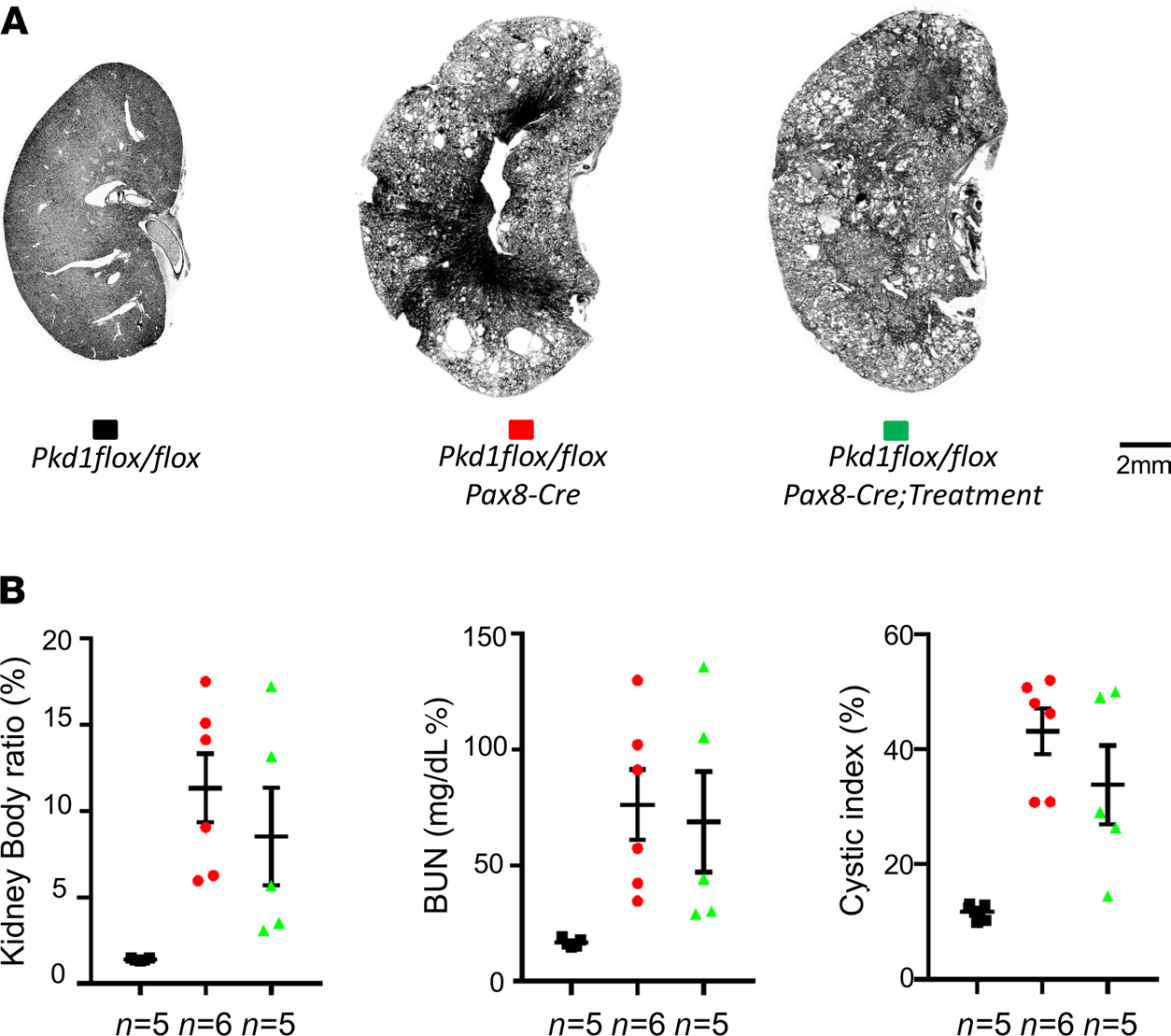
Effect of the dopamine receptor antagonist SCH2339 on renal cysts progression in *Pkd1^–/–^* mice. Inhibition DA1 receptor by SCH23390 has no effect on cyst progression in adult-onset autosomal dominant polycystic kidney disease model with selective inactivation of *Pkd1*. (**A**) Images of representative kidneys from adult-onset KO model (*Pax8 rtTA;Teto-Cre*) with the indicated genotypes at 18 weeks. (**B**) Aggregate data of the kidney weight/body weight ratio, cystic index, and BUN from the indicated number of mice. The color blocks in **A** correspond to the data in **B**. The numbers of animals (*n*) in each group are indicated below the histogram bars. Multiple group comparisons were performed using 1-way ANOVA followed by Tukey’s multiple comparison test and are presented as the mean ± SEM.

**Figure 7:**
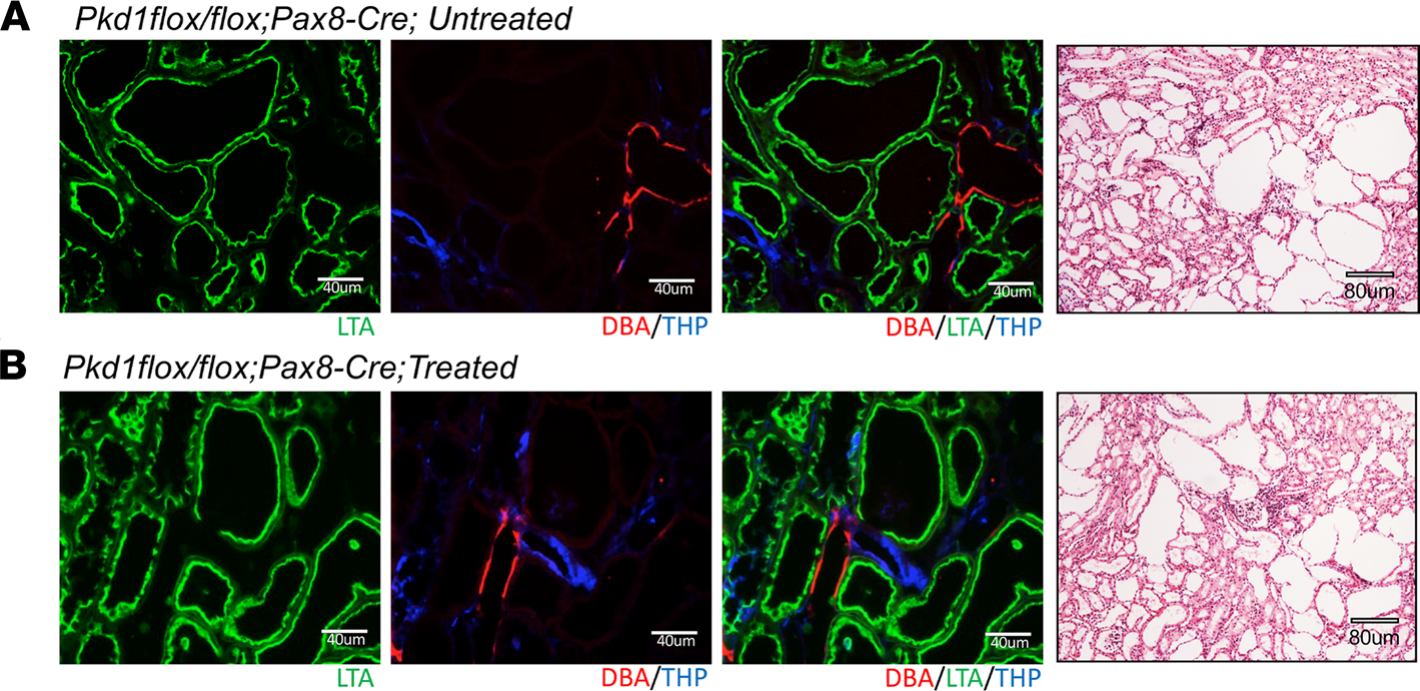
Kidney slices with HE staining and IF staining. IF with Rhodamine-conjugated Dolichos biflorus agglutinin (DBA) (red), FITC-LTA (green), and Sheep anti–tamm horsfall glycoprotein antibody (blue). Kidney sections were from 18-week-old adult *Pkd1^fl/fl^;Pax8-rtTA;TetO-cre* mice and SCH23390-treated *Pkd1^fl/fl^;Pax8-rtTA;TetO-cre* mice that had received doxycycline induction from P28 to P42.

Because SCH23390 blocked the DA1 receptor and also blocked dopamine-stimulated adenylate cyclase (26), inhibition of cAMP signaling may reduce cyst formation (27). To further investigate whether SCH23390 reduction in cyst formation may relate to its impact on flow-dependent transport, we studied the effect of SCH23390 on cyst formation in *Pkd1^–/–^* in which flow effect is intact. Similar experimental procedures were applied to noncyst control (*Pkd1^fl/fl^*) and *Pkd1^–/–^* (*Pkd1^fl/fl^;Pax8-rtTA;Tet-O-Cre* after 2 weeks induction by doxycycline from p28 to p42) with or without treatment with SCH23390. As shown in Figure 6, the kidney image, kidney weight/body weight ratio, BUN, and cystic index had no significant reduction between treated and untreated *Pkd1^–/–^* mice. Supplemental Figure 2 shows kidney images from all kidney histological sections used in Figure 6 that indicate no difference between treated and untreated cystic kidneys. Figure 7 shows kidney slices with H&E and IF staining using segmental-specific markers of LTA, THP, and DBA for identifying PT, LH, and collecting duct. H&E staining documents the similar extent of dilated tubules and cysts in both untreated and treated *Pkd1^–/–^* kidney sections. IF staining with segment-specific markers indicated that dilated tubules and cysts exist in all segments of kidney tubules, and SCH23390 did not change cyst distributions. We further performed power calculations using STPLAN (ver. 4.5; University of Texas, M.D. Anderson Cancer Center, Houston, Texas, USA) to investigate the possibility of SCH23390 also having an effect on *Pkd1*-KO mice with an increase in the number of treated mice. The results indicated that a minimum number of 720 mice would be needed for each group to obtain significant difference on BUN, kidney body weight ratio, and cystic index between control and treated groups in *Pkd1*-KO mice, as we demonstrated in *Pkd2*-KO mice. This result confirmed that *Pkd2* and *Pkd1* behave differently with respect to SCH23390 sensitivity.

### Mathematical model of hydrostatic pressures within the kidney.

**Figure 8:**
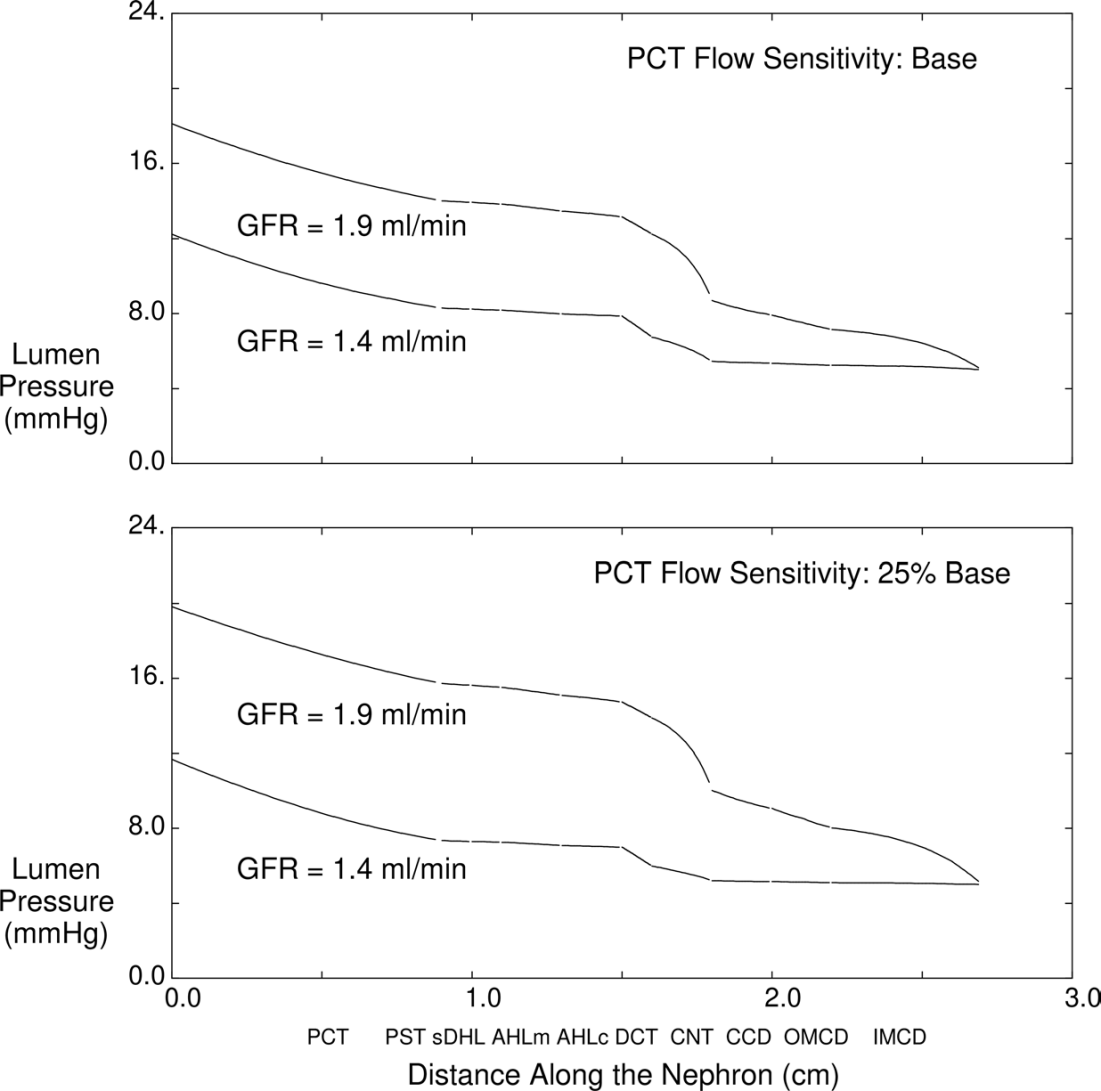
Model calculations of the impact of changes in GFR on tubule press. The abscissa is the distance along the superficial nephrons, with the first 9 mm corresponding to proximal convoluted tubule. The next 4 mm include proximal straight tubule, short descending Henle limb, and medullary ascending Henle limb. The next 3 mm are cortical ascending Henle limb and distal convoluted tubule. Then, there is a 2 mm connecting segment and, finally, 9 mm of the collecting ducts, from cortex to papilla. Both panels show luminal pressure along superficial nephrons for whole kidney when GFR is 1.4 mL/min and also for a 35% increase in GFR. In the upper panel, proximal convoluted tubule flow sensitivity is maintained at baseline; in the lower panel, the sensitivity of velocity-dependent Na^+^ reabsorption is reduced by 75%.

In Figure 8 and Table 5, calculations use a kidney model to examine the impact of changes in GFR on tubule pressures. In Figure 8, both panels show luminal pressure along superficial nephrons for the whole kidney when GFR is 1.4 mL/min and also for a 35% increase in GFR. In the upper panel, proximal convoluted tubule flow sensitivity is maintained at baseline; in the lower panel, the sensitivity of velocity-dependent Na^+^ reabsorption is reduced by 75%. For each curve, the largest pressure drops occur in the relatively long proximal convoluted tubules and in the connecting segments, which lose luminal area as they coalesce from 36,000 nephrons to 7200 cortical collecting ducts. It is only in the case of higher GFR, with greater urine flow, that there is any sizable pressure drop along the collecting duct system. The salient observation from this figure is that, with the increase in GFR, tubular pressure goes up; with weaker GTB, this pressure increase is greater. Table 5 shows the pressures and flows that correspond to these 4 model calculations. When glomerular filtration is normal, the 2 models (baseline and reduced PT flow–dependent transport) predict nearly identical nephron pressures. With the increase in GFR and urine flow, both models show higher pressures, and the pressure rise with weaker GTB is about 2 mmHg higher. Of note, the increase in nephron pressure with greater flow is attributable to the increase generated by collecting duct hydraulic resistance. The difference in pressure from early PT to the connecting segment of about 6 mmHg is preserved with each parameter set. In the lower half of Table 5 are simulations using the nephron model (28), in which the only compliant segments are the PT and in which the medullary interstitial conditions are unchanged from presumed antidiuretic values. With this model, the missing line in the table corresponds to the case of baseline glomerular filtration when proximal flow dependence is reduced. Under this condition, the model does not converge to a solution, since distal delivery of tubular fluid is too low to sustain tubular flow along the full nephron. For this nephron model, all urine flow rates are lower than in the kidney model, due to the hyperosmolar medulla. In the absence of strong proximal GTB, the increase in distal pressure is exaggerated.

## Discussion

The physiological importance of flow-dependent PT transport, or GTB, has been recognized for its role in the maintenance of body fluid and electrolyte homeostasis for more than 4 decades (1, 2). In mathematical models of rat kidney, increases in GFR increase intratubular hydrostatic pressures along the nephron; with impaired GTB, these increases in tubule pressure are more severe, in both proximal and distal segments. Mathematical models of the kidney have suggested that GTB may also mitigate swings in renal tubular hydrostatic pressure. In this study, we find that — prior to cyst formation — flow-dependent transport is absent in PT from *Pkd2^–/–^* mice and that flow dependence can be restored by a dopamine antagonist. In *Pkd2^–/–^* mice aged to allow for cyst formation, chronic administration of an oral dopamine antagonist slowed renal cyst formation; this did not occur in *Pkd1^–/–^*, which retained intact GTB in the precystic state. Our findings suggest that there may be differences in therapeutic approach to the 2 forms of PKD. Based on our examination of *Kif3a*^–/–^ tubules, we also demonstrate that cilia are not a prerequisite for GTB.

Multiple mechanisms may be involved in renal cyst formation in PKD. It has been found that inactivation of *Pkd1* or an essential IFT gene, tg737 (IFT88) in adult mice results in no cyst formation until months after deletion of either gene (29, 30). Although deletion of *Pkd1*, *Pkd2*, or *KIf3a* (required for cilia formation) produces renal cysts, with progressive enlargement of fluid-filled renal cysts when the disease reaches the late stage, different mechanisms are involved in cystic development and formation with *Pkd1*, *Pkd2,* and *Kif3a* mutations that may be due to differences in localization and functions within in the kidney (20). However, one similarity is that *Pkd1*, *Pkd2,* and Kif3a are all implicated in mechanosensory or chemosensory functions (10). *Pkd1* and *Pkd2* are expressed in primary cilia, and primary cilia play an important role in mechanosensory function responding to flow-induced changes of calcium signals in MDCK cells and in the cortical collecting duct (4, 16, 17). Whether *Pkd1* and *Pkd2* or primary cilia have a functional role of flow sensing mechanism in regulation of GTB in the PT has never been studied. Because complete genetic KO of *Pkd1* or *Pkd2* in mice results in embryonic lethality (22), we have used mice models of conditional inactivation *Pkd1* (*PKd1^fl/fl^;Pax8-rtTA;Tet-O-Cre*), *Pkd2* (*PKd2^fl/fl^;Pax8-rtTA;Tet-O-Cre*), and *Kif3a* (*Kif3a^fl/fl^;Pax8-rtTA;Tet-O-Cre*) (20, 31). Age-matched mice of these KO mice at the precystic stage were used to measure flow-activated Na and HCO_3_^–^ absorption in PT by microperfusion. In addition, the age-matched WT and Pkd2 heterozygous mice (22) were also used for the microperfusion study. Experimental data show that the flow-stimulated Na^+^ and HCO_3_^–^ absorptions are preserved in *Pkd1* (*Pkd1^fl/fl^;Pax8-rtTA;Tet-O-Cre*) and Kif3a (*Kif3a^fl/fl^;Pax8-rtTA;Tet-O-Cre*) mice, indicating that neither *Pkd1* or primary cilia are critical for flow sensing in PT. This result also suggests that neither intact PC1 nor the primary cilia are required for mechanosensory function in the PT. Our observed dissociation of flow-dependent transport and intact cilia in PT (Kif3a experiments) is consistent with findings in distal nephron segments, specifically preservation of flow-dependent Na^+^ reabsorption in the deciliated cortical collecting duct (32) and failure to observe flow-dependent Ca^2+^ responses in superfused inner medullary collecting ducts (33).

Experimental data demonstrate the absence of flow-stimulated Na^+^ and HCO_3_^–^ in both *Pkd2^+/–^* and *Pkd2^–/–^* (*Pkd2^fl/fl^;Pax8-rtTA;Tet-O-Cre* Pax8-rtTA) mice. The findings of this study suggest that the role of PC2 in flow-dependent transport derives from its functions outside the primary cilium. PC2, formerly TRPP2 encoded by *Pkd2*, is a calcium-activated cation channel expressed in the primary cilia, as well as in other compartments of the cell, including the endoplasmic reticulum (ER) (11). Previous studies showed that *Pkd2* mutation or KO resulted in an absence of IP3 receptor–mediated calcium release from the ER, indicating that PC2 plays an important role in IP3 receptor–mediated calcium signaling (12). We have demonstrated that IP3 receptor–mediated intracellular Ca^2+^ signaling plays a critical role in transduction of microvillus torque to increase Na^+^ and HCO_3_^–^ absorption in PT (21). The new observation of absence of GTB in *Pkd2^+/–^* and in *Pkd2^–/–^* mice could further derive from the functional role of *Pkd2* as the IP3 receptor–mediated TRPP2 channel in the ER (11). Alternatively, we have shown that intact cytoskeletal function is a prerequisite for flow-dependent transport in PT (3, 5). It is also known that there are structural connections between PC2 and cytoskeletal components (34). It is possible that the absence of PC2, or the presence of defective PC2, may compromise the signal transduction function of the actin cytoskeleton. If this were the mechanism underlying our observations, one might not see a functional difference between the *Pkd2* homozygote and heterozygote.

Motivation for these studies was derived, in part, from model calculations in a model of rat nephrons (28). In that model, a full ensemble of superficial and juxtamedullary nephrons fed into connecting segments, which coalesced into the collecting duct system. It was observed that variation in PT glucose reabsorption augmented or reduced distal flow, which in turn produced parallel changes in tubule pressures along the whole nephron, extending back to early PT (28). In that model, the PT was compliant, and proximal Na^+^ reabsorption was directly dependent upon luminal fluid velocity (23). Increased PT pressure with greater distal delivery, which has support in micropuncture observations in rat (35), found that acetazolamide increased LH delivery by 44% and urine flow 5-fold; with that, PT pressure increased from 13.6 to 15.6 mmHg. Those nephron model calculations suggested that, with defective flow-dependent proximal Na^+^ reabsorption, normal episodic increases in GFR might provoke larger swings in tubule pressure. It was speculated that such increases in tubular hydrostatic pressure might contribute to the creation and growth of renal cysts, analogous to the role of arterial pressure in creating vascular aneurysms.

The hypothesis generated by the nephron model was subject to at least 2 limitations: (a) medullary composition and pressure had been assigned so that interstitial pressure changes were not computed and (b) the only compliant nephron segments of that model were the proximal convoluted tubules. The first concern was addressed with creation of a kidney model, which included the medullary vasculature and, thus, enabled calculation of medullary interstitial conditions (36). Tubule compliance along all segments was introduced in a revision to that model (37). It must be acknowledged that the experimental data guiding assignment of tubule compliance were scant, with few studies in rat PT (38–40) and 1 regarding rat distal tubule (38). Nevertheless, in the kidney model’s simulation of a 35% increase in glomerular filtration, a 75% reduction in proximal flow sensitivity produced a 30% increase in both proximal and distal pressures (Table 5). In the noncompliant nephron model, the increase in tubule pressures was about 50%. These model observations provided the rationale for examining the dopamine antagonist SCH23390, which had been shown to enhance PT flow–dependent Na^+^ reabsorption (8), in order to see whether it might mitigate cyst growth in PKD.

Dopamine is the most important natriuretic signal to PT (41, 42). It acutely decreases surface NHE3 expression in opossum kidney (OK) cells and stimulates NHE3 endocytosis in PT (24). These effects depend on the DA1 receptor– and DA2 receptor–mediated protein kinase A (PKA) (24). A prior study of the perfusion of isolated rabbit tubules reported that dopamine had no effect on PT transport in unstimulated conditions but abolished the norepinephrine-induced increase in Na^+^ absorption (25). This observation is consistent with our data showing that dopamine had no effect on NHE3 activity at low flow rates but abolished the flow-stimulated Na absorption and partially inhibited the flow-stimulated HCO_3_^–^ absorption (8). In this study, we provide evidence that impaired GTB can occur before renal cystic formed and increased the tubule sensitive to flow by a DA1 antagonist, slowing the renal cyst formation in *Pkd2* KO mice. In the case of *Pkd1* or *Kif3a* mice, there was no abnormality in flow-dependent regulation of PT transport, and SCH23390 had no salutary effect on cyst formation. It must be acknowledged that this study has not provided proof that the cyst protection afforded by SCH23390 derived from its effect on PT, just that its PT action motivated our decision to try it as a therapeutic. It must also be acknowledged that we have not provided measurement of the critical variable in the hypothesis that episodically elevated tubule hydrostatic pressures play pathophysiologic roles in the cyst formation of *Pkd2*. At this point, it is not clear how such data could be obtained.

Common pathogenic mechanisms of the Autosomal Dominant Polycystic Kidney Disease (ADPKD) — caused by mutations in *Pkd1*or *Pkd2*, including disruption of intracellular calcium homeostasis, excessive cell proliferation and fluid secretion, abnormal extracellular matrix, and disruption of mechanisms controlling tubular diameter — contribute to cyst formation (43). There was no specific difference in these common pathogenic mechanisms for *Pkd1* or *Pkd2* mutation–caused ADPKD. Our study suggests a potentially new pathogenic phenotype of *Pkd2* deletion — namely, a pattern of chronic episodic tubule dilatation in response to swings of luminal hydrostatic pressure.

## Methods

### Mouse husbandry and doxycycline induction.

*Pkd1^fl/fl^*, *Pkd2^fl/fl^*, *Kif3a^fl/fl^; Pax8-rtTA*, and *TetO-cre* mouse lines have been described previously (20). Since all 3 animal models are well characterized, the methods of produce control and KO mice were carefully followed as described previously (20). Mouse lines in this study that used Pax8-rtTA; TetO-cre transgenic system for gene deletion were administered doxycycline for 2 weeks, beginning at P28 for adult induction. Doxycycline solution was made from drinking water supplemented with 2 mg/mL doxycycline hyclate (D9891; MilliporeSigma) and 3% sucrose (S-0389; MilliporeSigma). Animals treated at P28 were switched from regular drinking water to doxycycline solution for 2 weeks and then returned to regular drinking water until the study end point.

At the study end points, mice were euthanized, and kidneys and serum were collected as described previously. *Pkd2* was inactivated by inserting a selectable *neo^r^* cassette into the *Not*I site at codon 59 in exon 1 in the same transcriptional orientation as *Pkd2* (22).

### Microperfusion of kidney PT.

Eight- to nine-week-old mice (male and female) were used for microperfusion in vitro. A standard method for isolated tubule perfusion was used as described previously (5). Briefly, animals were anesthetized with i.p. sodium pentobarbital 100 mg/kg, and freshly dissected PT were perfused with an ultrafiltrate-like solution containing (in mM) 125 NaCl, 25 NaHCO_3_, 1 CaCl_2_, 1.2 MgSO_4_, 2 glutamine, 2 lactic acid, 5 glucose, 5 KCl, and 1.2 phosphoric acid. Extensively dialyzed [^3^H]-methoxy-inulin was added to the luminal perfusate at a concentration of 30 μCi/mL as a volume marker. The bath solution contained similar electrolytes as the luminal solution, with added 3 g/dL albumin. The perfusate and bath solutions were bubbled with 95% O_2_–5% CO_2_, the pH was adjusted to 7.4, and the osmolalities to 300 mosmol/KgH_2_O in both solutions. Bath fluid was continuously changed at a rate of 0.5 mL/min to maintain the constancy of pH and bath osmolality. PT were perfused at either low (5 nL/min) or high (20 nL/min) perfusion rates, and the tubular fluid was collected (5). Three timed collections of tubular fluid were made, and ^3^H concentrations and total CO_2_ concentrations in perfusate and collected sample fluid were measured; rates of fluid, Na^+^ and HCO_3_^–^ absorption were calculated by standard methods (5). The J_Na_ was calculated according to the rate of fluid absorption ([Na] × J_v_), since the ratio of fluid and Na^+^ absorption is 1 in the PT (3).

### IF and imaging.

Mice were sacrificed at age of 18 weeks, and kidneys were fixed in 4% PFA for histological analysis. Sagittal sections of kidneys were processed for H&E staining.

Renal markers used for IF were Rhodamine-conjugated DBA (1;1000 dilution, Vectors Laboratories), FITC-LTA (1;1000 dilution, Vectors Laboratories), mouse anti-calbidin (1:1000, MilliporeSigma), and sheep anti–THP glycoprotein antibody (1;1000, Bio-Rad). Images were taken on a Nikon Eclipse TE2000-U microscope driven by MetaMorph software (Universal Imaging).

### Cystic index calculation and BUN measurements.

Plasma samples were collected from cardiac puncture, and kidneys were harvested in control, *Pkd1*, and *Pkd2* KD mice at the age of 18 weeks for BUN and cyst analysis. The extent of tubular cyst formation was quantified in sagittal sections of whole kidneys. Four sections (2 each from the midsagittal region of each kidney) were analyzed for each experimental animal. Whole kidney images were obtained using automated image acquisition by the scan slide module in MetaMorph (Universal Imaging). Total kidney area, total cystic area, and total noncystic area were measured using the integrated morphometry feature in MetaMorph. Cystic index = (total cystic area/total kidney area) × 100 and is expressed as a percent (31). Plasma BUN values were determined spectrophotometrically with a diacetylmonoxime-based assay kit (Stanbio Laboratory) by Yale George M. O’Brien Kidney Center.

### Statistics.

Data are presented as mean ± SEM. Two-tailed Student’s *t* test was used to compare control and experimental groups. One-way ANOVA was used for comparison of several experimental groups with a control group, followed by a Tukey’s multiple comparison. The difference between the mean values of an experimental group and a control group were considered significant if *P* < 0.05.

### Study approval.

Experiments were carried out under protocols approved by the Yale University Institutional Animal Care and Use Committee in accordance with NIH guidelines for the ethical treatment of animals.

## Author contributions

ZD performed microperfusion experiments, analyzed experimental results, and summarized data in Table 1, Table 2, Table 3, and Table 4 under the supervision of TW. XT and MM performed genotyping and induced *Pkd1*, *Pkd2*, and *Kif3a* cKD mice under the supervision of SS. TW preformed experiments with chronic treatment of *Pkd1^–/–^* and *Pkd2^–/–^* with DA1 antagonist. XT performed experiments of cyst analysis, IF, and imaging under the supervision of SS. AW performed mathematical model analysis. TW and AMW designed experimental approaches and draft the manuscript. All authors were involved in revising the manuscript for intellectual content. All authors read and approved the final manuscript.

## Supplementary Material

Supplemental data

## Figures and Tables

**Table 5:**
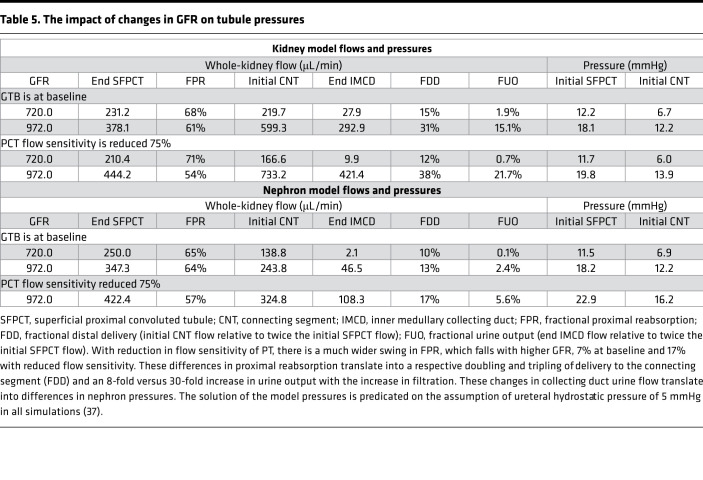
The impact of changes in GFR on tubule pressures

**Table 4:**
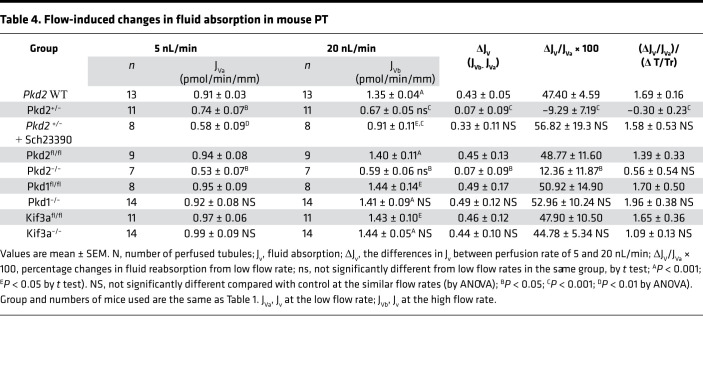
Flow-induced changes in fluid absorption in mouse PT

**Table 1:**
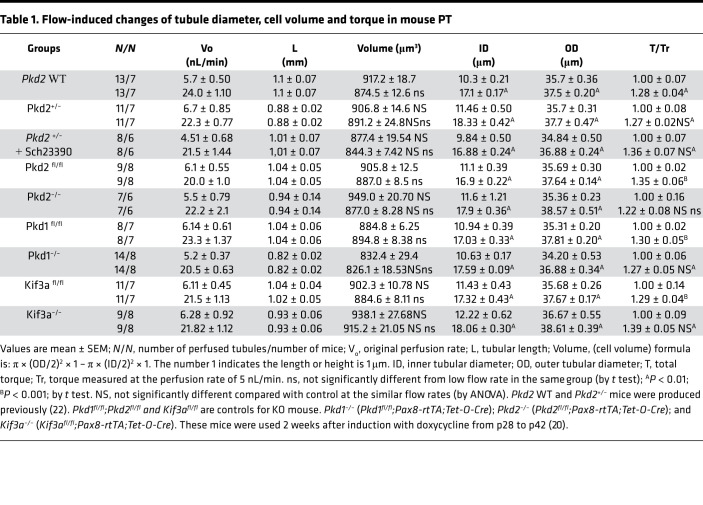
Flow-induced changes of tubule diameter, cell volume and torque in mouse PT

**Table 2:**
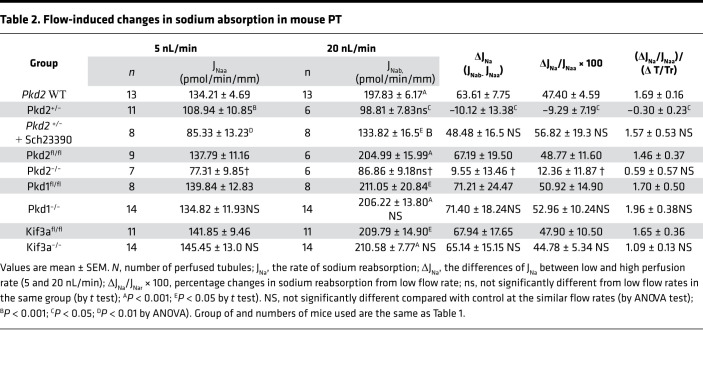
Flow-induced changes in sodium absorption in mouse PT

**Table 3:**
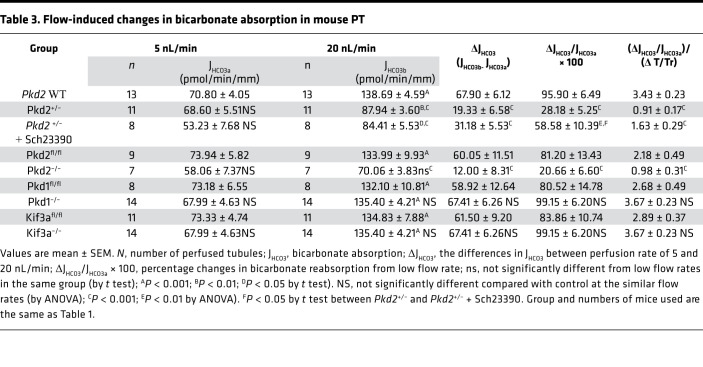
Flow-induced changes in bicarbonate absorption in mouse PT

## Version 1 04/22/2021

In-Press Preview

## Version 2 05/24/2021

Electronic publication

## Version 3 05/24/2021

Corrected typographical error in Figure 5 legend.
