# Supplementary material for: Restoration of proximal tubule flow–activated transport prevents cyst growth in polycystic kidney disease
Source: JCI Insight. 2021 May 24;6(10):e146041. doi: 10.1172/jci.insight.146041 (PMC8262298; doi:10.1172/jci.insight.146041)
Supplement: Supplemental data [file jciinsight-6-146041-s184.pdf]

Supplemental Materials:

Supplemental Materials include two additional Figures.

Supplementary Figure 1. Images of all kidney histological sections used in Fig. 4A.

Supplementary Figure 2. Images of all kidney histological sections used in Fig. 6A.

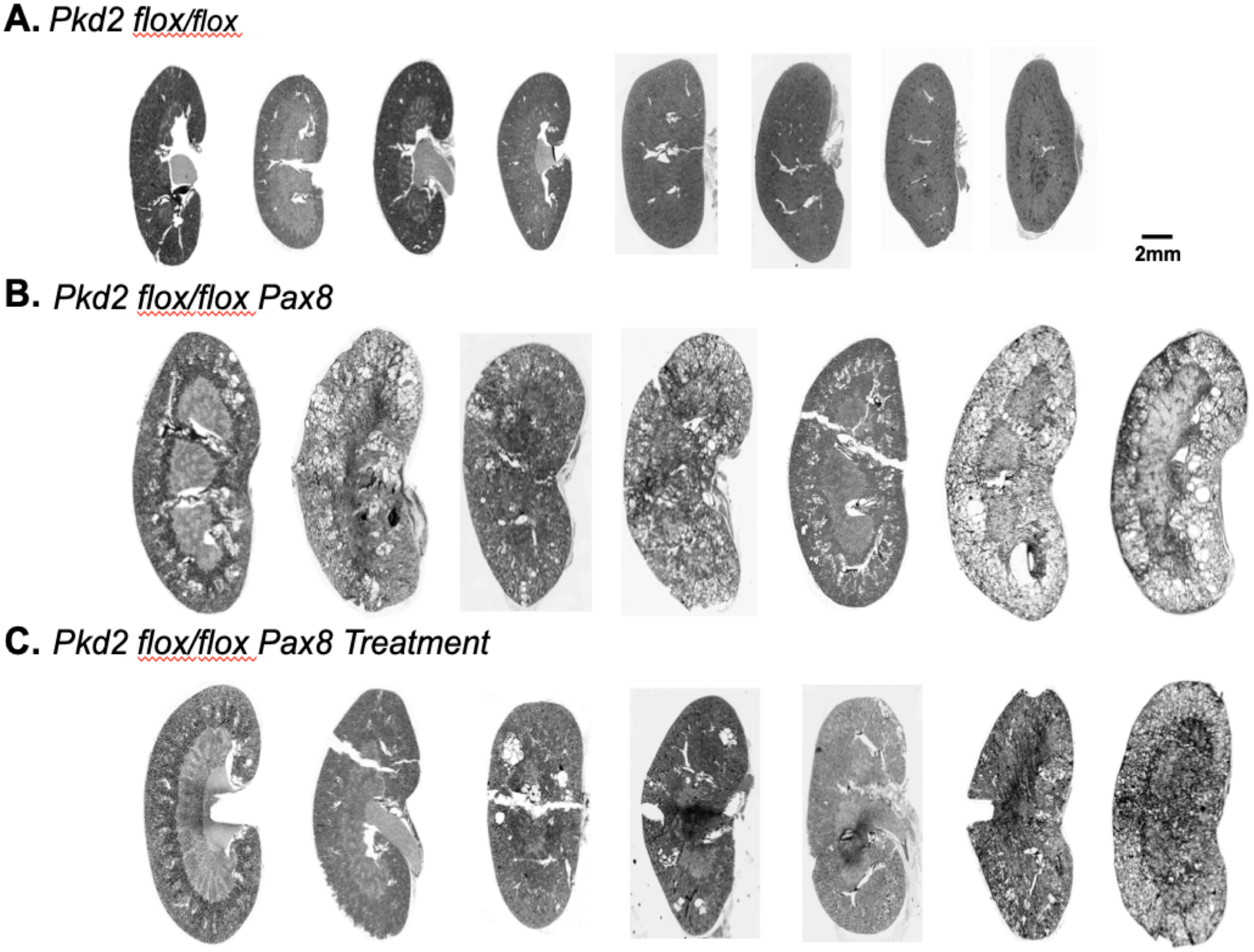

Supplementary Figure 1. Images of all kidney histological sections used in Fig. 4A.

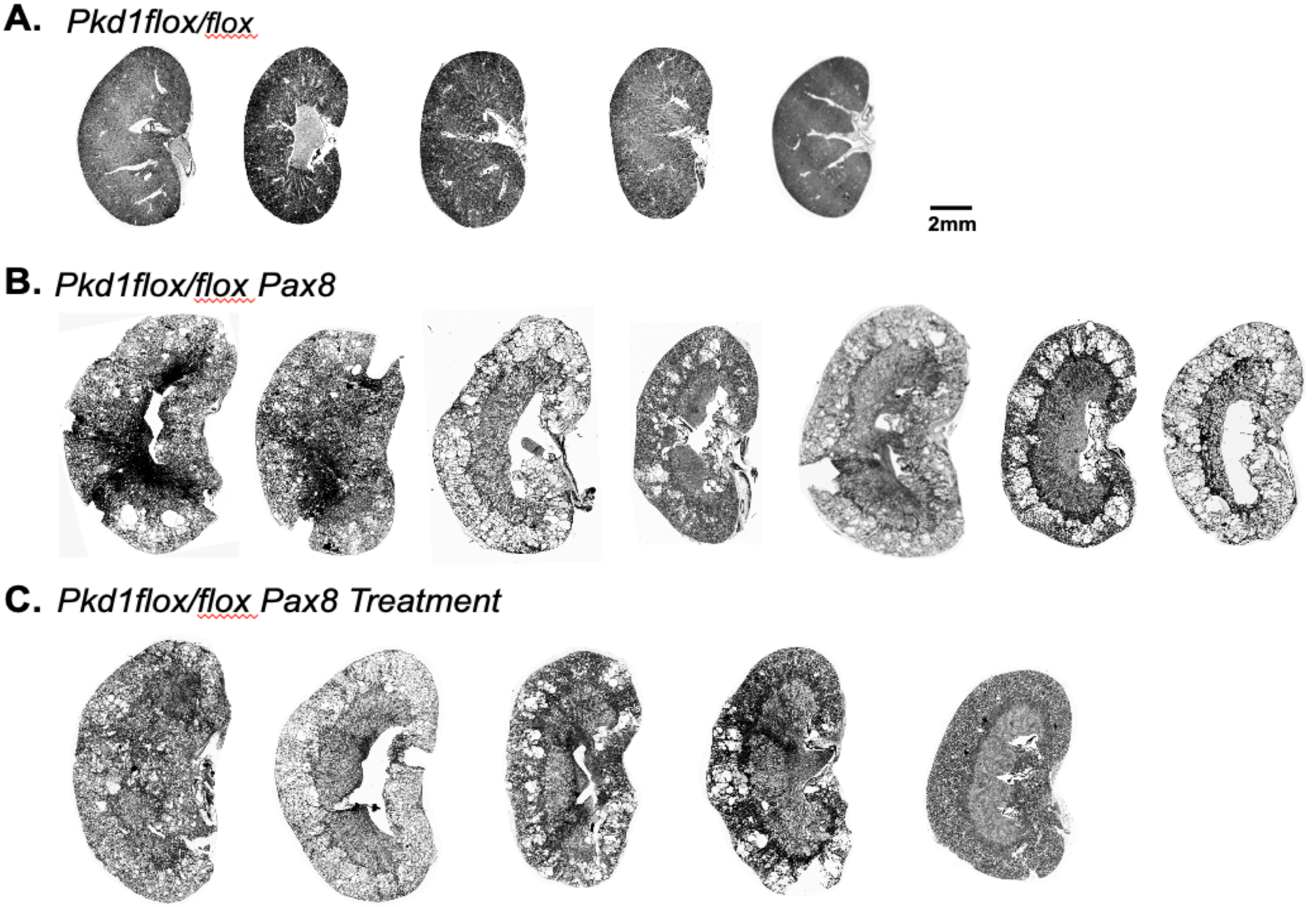

Supplementary Figure 2. Images of all kidney histological sections used in Fig. 6A.
